# Books and Magazines

**Published:** 1901-01

**Authors:** 


SAUNDERS POCKET MEDICAL FORMULARY.—With an Appendix containing posological table; formulas and doses for hypodermic medication; poisons and their antidotes; diameters of the female pelvis and fœtal head; obsterical table; diet list for various diseases; materials and drugs used in antiseptic surgery; treatment of asphyxia from drowning; surgical remembrances; tables of incompatibles; eruptive fevers; weights and measures, etc. By Wm. M. Powell, M. D., author of "Essentials of diseases of children;" member of the Philadelphia Pathological Society, etc. Sixth edition. Thoroughly revised. Pp. 298. Price, $2.00. Philadelphia: W. B. Saunders & Co., 925 Walnut Street, 1900.

A Text Book of Special Surgery.—For practitioners and students. Translated from the seventh German Edition, which has but recently appeared. By Arthur B. Hosmer, M. D., and edited by Christian Eenger, M. D.

The publishers, Herbert S. Stone & Co., Eldridge Court, Chicago, announce that they have in preparation and will shortly issue the above mentioned important work. It is the authorized translation and will consist of three large octavo volumes, on an especially fine grade of plate paper, and each volume will contain in the neighborhood of three hundred illustrations. The price is not announced. Interested readers are referred to the publishers for the price and further particulars.

"Panama and the Sierras: A Doctor's Wander Days."—By Dr. G. Frank Lydston, Chicago. The Riverton Press, Chicago. Cloth: Handsomely gotten up and in first-class style, beautifully illustrated from "snap shots" by the author. 283 pages. Clear type, tony paper, etc.; and all for $1.75, postpaid.

Reading this book gives one an appetite, and but for the grewsome incident and picture of a California rope party, would leave a good taste in the mouth. It is more like drinking champagne than anything in my experience. It has all the bubble, sparkle and snap of "Mumms," but is not at all *"dry."* Or, it may be likened unto a babbling brook"—which dodges now and then into cool, shady places, but for the most part rattles along in the sunshine and scatters its rays all about—little "glints" and big "glints." There is but one Lydston.

MEDICINE AS A BUSINESS PROPOSITION.*—By Frank G. Lydston, M. D., Professor of the Surgery of the Genito-Urinary Organs and Syphilology, in the Medical Department of the State University of Illinois; Professor of Surgery, Chicago Clinical School. *Lecture delivered at the public meeting of the St. Joseph County Medical Society South Bend, Indiana, January 30, 1900.

"So live that when thy time has come to join the innumerable caravan which moves to that mysterious bourne peopled by doctors who have died of innutrition, thou go not like the general practitioner called at night, scourged from his office, but, sustained and soothed by the motto, "Never trust," approach thy grave like one who wraps his stocks and bonds about him and lies down to pleasant dreams." (With apologies to the shade of W. C. B.)

Dr. Lydston's admirable lecture is published in pamphlet form of 32 pages. It is intensely interesting reading, and is in a style peculiarly Lydston's own. He handles the subject ably, and vigorously, and while there are some things in it which we do not approve, still in the main he is right, medicine is no longer a philanthropic "mission"—in the exercise of which one is expected to be too modest to make a charge, but must take an *honorarium*—whatever is offered—if any is offered. Medicine is a business, whereby one hopes and expects and endeavors to make a living at least, perhaps more. But many go to the other extreme and resort to questionable means—to get patients. The doctor—I mean the capable and meritorious doctor—invests his money—puts in some of the best years of his life, and does some very hard and disagreeable work to acquire such knowledge as will make his services "worth while" and give them a money value. Yet one who has invested neither time, money nor labor in the acquisition of knowledge and who really has none worth mentioning—but has assurance —and other admirable winning qualities—will open up next door to him and take away all his bread and butter and automobiles and things by means which no reputable doctor would stoop to. When Lydston turns loose all holds and goes for these fellows it rings like hammer on anvil and makes the fire fly.

